# Meckel's diverticulum containing enterolith mimicking acute appendicitis

**DOI:** 10.1016/j.ijscr.2022.107497

**Published:** 2022-08-10

**Authors:** ZakaUllah Jan, Nisar Ahmed, Naila Aziz, Maliha Tariq, Danish Khattak, Muhammad Sohaib Asghar

**Affiliations:** a Khyber Teaching Hospital, Peshawar, Pakistan; b Dow University of Health Sciences-Ojha Campus, Karachi, Pakistan

**Keywords:** Meckel's diverticulum, Enterolith, Case report, Acute appendicitis

## Abstract

**Introduction:**

Meckel's diverticulum is a vestige of the vitello-intestinal duct. It is one of the most common congenital abnormality of the GI tract.

**Case presentation:**

We present a case of a male patient who presented with pain in the peri-umbilical region and in right iliac fossa. A clinical diagnosis of acute appendicitis was made, but, interestingly it turned out to be a Meckel's diverticulum having enterolith intraoperatively. The patient underwent wedge resection and the post-operative course was uneventful.

**Conclusion:**

Meckel's diverticulum containing enterolith is rare. This can mimick as acute appendicitis and the surgeon needs to be wary of this.

## Highlights

• Meckel's diverticulum containing enterolith is rare.
• This can present as an episode of acute appendicitis.
• One needs to have a high index of suspicion for Meckel’s diverticulum in such cases.
• Diagnostic Laparoscopy plays a pivotal role in such cases.
• Segmental resection is the treatment of choice.

## 1 Introduction

Meckel's diverticulum is one of the most common congenital anomaly of GI tract, observed in about 2 % of population [1]. It equally involves both genders, however male patients usually become symptomatic resulting from the epithelium contained in the meckel's [2].

Anatomically it is about 3–5 cm in length, commonly situated 45-60 cm from ileocecal junction, on the anti-mesenteric border of ileum [3]. It is supplied by a separate branch from superior mesenteric artery [4].

Microscopically it is similar to small bowel, containing all the three intestinal layers and may contain heterotrophic gastric, pancreatic or colonic mucosa which may cause other complications. Meckel's diverticulum is the vestige of vitello-intestinal duct which connects the embryo to the primitive gut [1]. It regresses within 5th–7th week of fetal life. Its failure to regress can cause various anomalies. It mostly remain silent and diagnosed incidentally during contrast studies or intraoperatively [5], [6], [7], [8], [9], but can present with gastrointestinal bleeding in 20–50 % of patients with complications [10], [11].

In our case, patient had signs and symptoms of acute appendicitis, which is one of the differentials for Meckel's diverticulum.

This work has been reported in line with the SCARE 2020 criteria [12].

## 2 Case presentation

A 40-year old male patient, known diabetic for last 6 years, presented to the outpatient clinic with a 1 day history of abdominal pain mostly in the right iliac fossa. Initially, the pain was at peri-umbilical, but then shifted to the right iliac fossa. He denied any history of constipation or any other surgical intervention. Pain was relieved with some analgesia but recurred after 1 to 2 h.

On clinical examination, his vitals were normal with a blood pressure of 130/80 mmHg & a pulse rate of 88 bpm. On abdominal examination, his abdomen was non-distended, and he was tender in the right iliac fossa. Obturator, rovsing's and psoas signs were positive.

His hemoglobin was 14 g/dl and total leukocytes count was 13 × 10^3^ cmm. There were no significant findings in his urine examination.

**Figs. 1 & 2:**
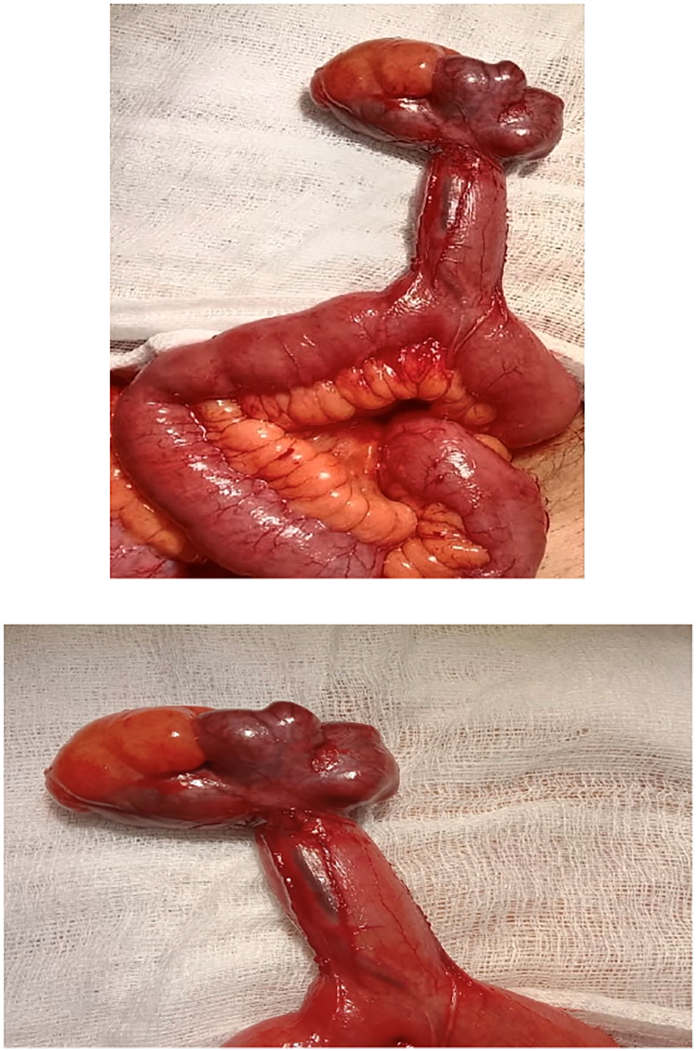
Showing Meckel's diverticulum and its feeding vessel.

**Fig. 3:**
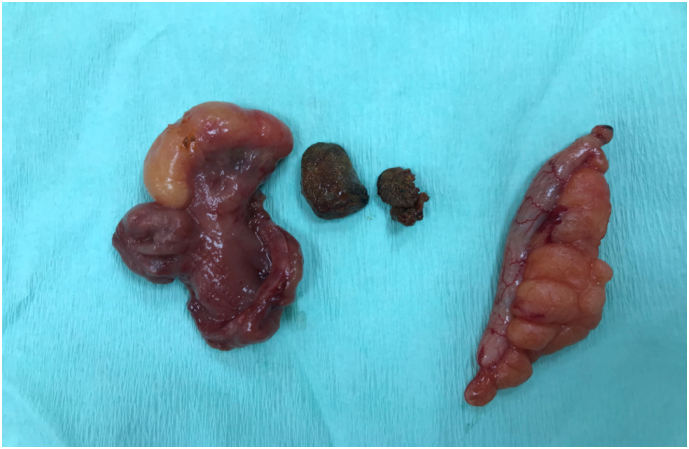
Showing the resected specimen, enterolith & the appendix.

A clinical diagnosis of acute appendicitis was made. Patients was prepared for surgery. Under General Anesthesia, diagnostic laparoscopy showed Meckel's diverticulum in addition to a mildly inflammed appendix. Decision was taken to convert the procedure to open by making a grid iron incision at the McBurney's point. On exploration, there was a huge meckel's diverticulum containing enterolith. Wedge resection of the Meckel's diverticulum was done by the consultant. Then appendectomy was performed. The patient made an uneventful recovery post-operatively (Figs. 1, 2, 3).

The patient was sent home on the 3rd day post-operative day. The stitches were taken out on the 10th post-op day.

**Fig. 4:**
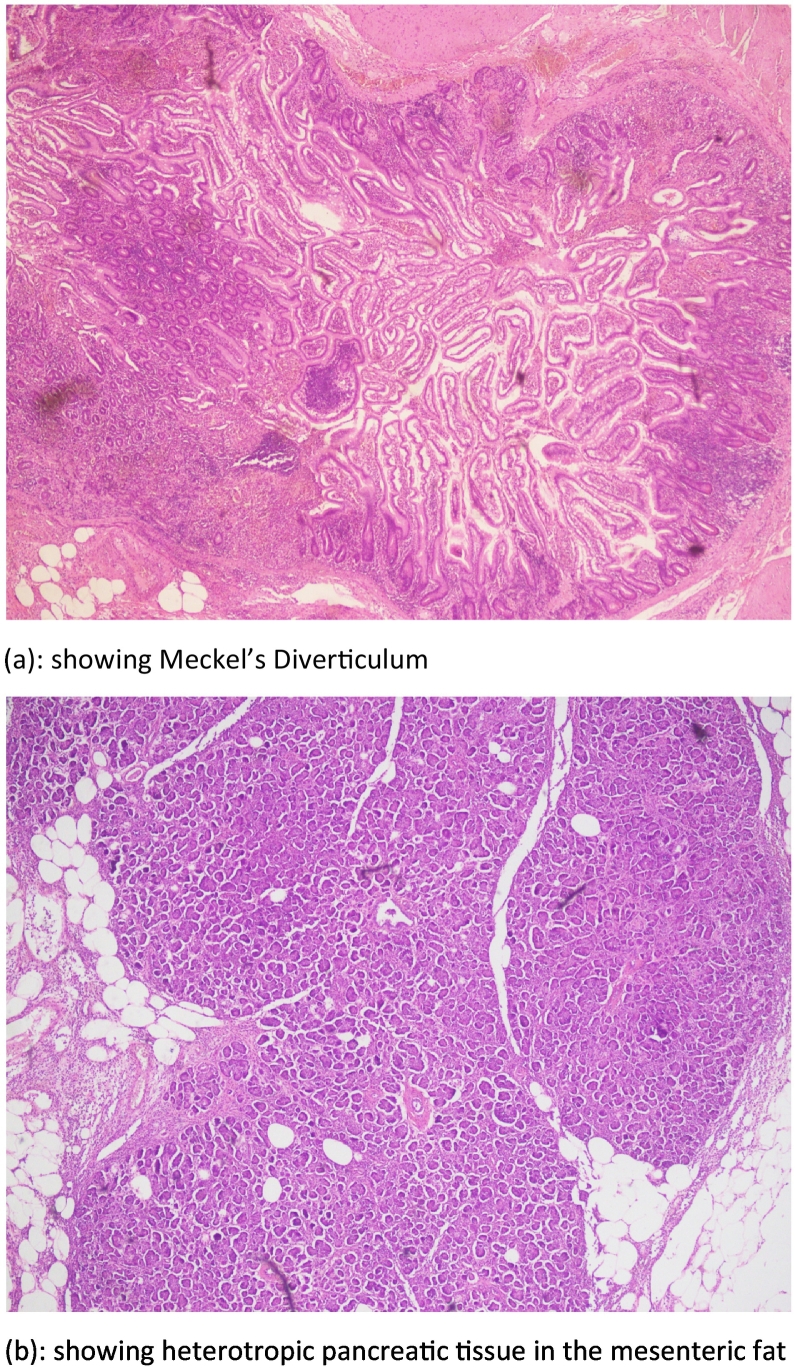
(a): Showing Meckel's diverticulum. (b): Showing heterotropic pancreatic tissue in the mesenteric fat.

On two weeks follow up, the histopathology revealed meckel's diverticulum with moderate acute and chronic inflammation, heterotropic pancreatic tissue in the mesentery of diverticulum & appendix with fecolith. There was no evidence of any malignancy [Fig. 4(a) and (b)].

## 3 Discussion

Meckel's diverticulum is one of the most common abnormal development of the GI system that results from a fragmentary vitelline canal. The diagnosis of Meckel's diverticulum may be difficult as the condition remains asymptomatic or may imitate varied ailments and blanket the clinical likeness. Life threatening complications can arise including bleeding, obstruction, inflammation and perforation. Thus, it's imperative that anatomical and pathophysiological characteristics are comprehended in order to preclude complications which will influence morbidity and mortality [13].

Meckel's diverticulum is similar to the small intestine and has all the three layers. Because it is supplied by its own artery, it's sensitive to infection. In 50 % of cases it contains ectopic mucosa which can be pancreatic or gastric in origin. Infrequently a colonic or hepato-biliary tissue is found. Gastrointestinal hemorrhage may occur as a result of ulceration in the gastric or the adjoining ileal mucosa. Therefore, if simple resection of the diverticulum is performed in such cases, bleeding will reoccur in post-operative period. Therefore, segmental resection is recommended in these cases [13].

Diagnosis is tricky and infrequently made, and imaging, especially in a complicated case is frequently not helpful; still diagnostic laparoscopy has an important role. The same was true in our case. Our patient also underwent diagnostic laparoscopy and that led us to the diagnosis of Meckel's diverticulum. The onset of complications decreases with age, and the diagnosis of Meckel's diverticulum in the grown-up is infrequent. In case of complications, resection is indicated; although controversial when Meckel's diverticulum is found incidentally. A large series in the literature shows that surgery is not indicated in the absence of factors responsible for complications; i.e. age younger than 40, male gender, the presence of mucosal changes observed at surgery and diverticulum longer than 2 cm. Resection and anastomosis is preferable to tangential mechanical stapling or wedge resection, because of the hazard of leaving behind aberrant heterotopic mucosa [14].

Majority of the Meckel's diverticulum never develop their characteristic feature, however complications like bleeding or perforation has given rise to the debate whether a silent MD should be resected if discovered by chance during the operation. To the best of author's knowledge, this query has yet to be answered. Moreover, with the advancements in laparoscopic surgery, & better imaging modalities like computed tomography (CT), the epidemiology of MD demands to be reassessed.

## 4 Conclusion

Meckel's diverticulum – A remnant of the vitello-intestinal duct, is one of the most common congenital abnormality of the GI tract. MD containing enterolith is rare. MD must be considered in the differentials of acute appendicitis and the surgeon must be wary of this.

## 5 Patient perspective

I was alright when suddenly one day I had pain around my umbilicus. I took over the counter pain killers that give me temporary pain relief. A few hours later, I again had an episode of the pain but this time I also noticed the pain was in my right flank region. I went to my colleague who is a surgeon. When he examined and investigated me, he told me that this is an episode of acute appendicitis and so I had to give him the go-ahead for the operation. Hence I was operated upon. But surprisingly I was told afterwards that in addition to a mildly inflamed appendix, I had a Meckel's diverticulum containing some sort of a stone which was causing all these symptoms. I was treated well in time and I am now alright. I thank the team at Surgical ‘A’ Unit, Khyber Teaching Hospital Peshawar who took really good care of me.

## Declaration of competing interest

There are no conflicts of interests.
